# PRKX down-regulates TAK1/IRF7 signaling in the antiviral innate immunity of black carp *Mylopharyngodon piceus*

**DOI:** 10.3389/fimmu.2022.999219

**Published:** 2023-01-11

**Authors:** Xiao Yang, Yue Ai, Liang Chen, Chanyuan Wang, Ji Liu, Jie Zhang, Jun Li, Hui Wu, Jun Xiao, Mingxian Chang, Hao Feng

**Affiliations:** ^1^ State Key Laboratory of Developmental Biology of Freshwater Fish, College of Life Science, Hunan Normal University, Changsha, China; ^2^ State Key Laboratory of Freshwater Ecology and Biotechnology, Institute of Hydrobiology, Chinese Academy of Sciences, Wuhan, China; ^3^ Key Laboratory of Hunan Province for Study and Utilization of Ethnic Medicinal Plant Resources, College of Biological and Food Engineering, Huaihua University, Huaihua, China

**Keywords:** black carp, PRKX, TAK1, IRF7, interferon, SVCV

## Abstract

TGF-β-activated kinase-1 (TAK1), tightly related to innate immunity, is phosphorylated and activated by X-linked protein kinase (PRKX) in humans and mammals, which belongs to the c-AMP-dependent protein kinase family. However, the relationship between PRKX and TAK1 remains unknown in teleost. It has been reported in vertebrates for the first time that TAK1 of black carp (bcTAK1) interacts with bcIRF7 and is capable to up-regulate bcIRF7-mediated IFN signaling in our previous study. In this study, the role of PRKX homologue of black carp (*Mylopharyngodon piceus*) (bcPRKX) in bcTAK1/IFN signaling has been explored. Overexpression of bcPRKX suppressed the transcription of interferon promoters but enhanced the transcription of NF-κB promoter. *Mylopharyngodon piceus* kidney (MPK) cells transfected with shRNA targeting *bcPRKX* gene presented enhanced antiviral activity against spring viremia of carp virus (SVCV), in which the mRNA levels of the antiviral proteins were increased, including MX1, Viperin and PKR. Overexpressed bcPRKX dampened bcTAK1/bcIRF7/IFN signaling in the luciferase reporter assay and plaque assay. The interaction between bcTAK1 and bcPRKX has been identified by the immunofluorescence (IF) staining and co-immunoprecipitation (co-IP) assay. In addition, we found that bcPRKX can trigger the degradation of bcTAK1. However, the lysosome inhibitor chloroquine, but not the proteasome inhibitor MG-132, prevented the bcTAK1 degradation mediated by bcPRKX. Thus, we conclude that bcPRKX inhibits bcTAK1/bcIRF7/IFN signaling during the innate immune activation by targeting bcTAK1 and triggers lysosome-dependent degradation of bcTAK1.

## Introduction

Vertebrates have developed powerful immune system to fight against invading pathogens, which is comprised of innate immunity and adaptive immunity (1). As the first line of host defense mechanism, the innate immune system recognizes pathogenic microbes, such as bacteria and viruses, through germ line-encoded pattern recognition receptors (PRRs) (2). PRRs recognize certain pathogen-associated molecular patterns (PAMPs) and transfer signal to downstream cascade (3), leading to the production of interferons (IFNs) and inflammatory cytokines finally (4–6).

IFNs are a family of induce-expressed cytokines that initiate a series of activities resisting viral infection (7, 8). Despite the difference in amino acid sequences and structure, all IFNs exhibit antiviral activity as well as anti-tumor ability, and some IFNs show involvement in adaptive immune response as well (7, 9, 10). Production of IFNs requires a number of transcription factors (11). Transcription of IFN-β, for instance, requires interferon regulatory factor 3 (IRF3), IRF7, nuclear factor-κB (NF-κB) and ATF-2/c-JUN to bind cooperatively to the enhancer of IFN-β, recruiting cofactors and chromatin-remodeling enzymes to the IFN-β promoter (12).

TGF-β (transforming growth factor β)-activated kinase-1 (TAK1) is among the important factors in NF-κB signaling and belongs to the mitogen-activated protein kinase kinase kinase (MAP3K) family. As a critical serine/threonine kinase, TAK1 has been well characterized by its essential role in TNF receptor (TNFR)-, IL-1 receptor I (IL-1RI)- and Toll-like receptors (TLRs)-mediated activation of NF-κB and mitogen-activated protein kinase (MAPK) (13–16). In addition, TAK1 has been suggested to take part in the activation of IRF3 and induction of a series of interferon-stimulated genes (ISGs) (17).

Neither NF-κB signaling deficiency nor dysregulation of NF-κB maintains homeostasis of cells (18–20). The X-linked protein kinase (PRKX), as an important regulator of TAK1, is a cAMP-dependent protein kinase belonging to the AGC kinase subfamily (21). Full activation of TAK1 needs several signals including phosphorylation of threonine 178, 184, 187 and serine 192, 412 (22–24). With another AGC protein kinase member protein kinase A (PKACα), PRKX phosphorylates TAK1 on serine 412, which is crucial for TAK1 response to proinflammatory stimuli (25). Furthermore, Rep52, one of four adeno-associated virus (AAV) encoded nonstructural proteins, was found to drastically inhibit PRKX kinase activity and Rep78 suppressed PRKX-activated CREB-dependent transcription (26, 27). As a cAMP-dependent protein kinase, PRKX takes part in multiple processes in cell growth and development. For instance, recent reports have revealed that PRKX is involved in female congenital vagina and uterus disease as well as functions in ovarian cancer development (21, 28–31). However, the mechanism behind PRKX in innate immunity is relatively less investigated. Compared with its mammalian counterpart, the role of teleost PRKX remains largely unknown.

In this paper, a PRKX homologue of black carp (bcPRKX) has been cloned and characterized. bcPRKX down-regulated IFN promoter transcription but enhanced NF-κB promoter transcription in the reporter assay. Knock-down and overexpression of bcPRKX verified its role in negative regulation of the antiviral immune response against spring viremia of carp virus (SVCV). bcPRKX suppressed bcTAK1/bcIRF7-mediated IFN induction as well as triggered lysosome-mediated degradation of bcTAK1, which provide new insights into a previously unrecognized role of PRKX in the homeostasis of innate immune signaling in vertebrates.

## Materials and methods

### Cells, plasmids, antibodies and reagents

HEK 293T cells, *Epithelioma Papulosum Cyprinid* (EPC) cells, *M. piceus* kidney (MPK) cells and *Ctenopharyngodon idella* kidney (CIK) cells were kept in the laboratory and these cell lines were cultured in DMEM (Gibco, USA) containing 10% fetal bovine serum (FBS), 2 mM L-glutamine, penicillin (100 u/mL) and streptomycin (100 μg/mL) (32). EPC, MPK, and CIK cells were cultured at 26°C with 5% CO_2_; HEK 293T cells were cultured at 37°C with 5% CO_2_.

Plasmids in this study were listed in **Table 1**, antibodies in **Table 2** and reagents in **Table 3**.

**Table 1:** Plasmids used in this study.

Plasmid	Application
Overexpression	
pcDNA5/FRT/TO-Flag-bcPRKX pcDNA5/FRT/TO-HA-bcPRKX pcDNA5/FRT/TO-Myc-bcPRKX pcDNA5/FRT/TO-Flag-bcTAK1 pcDNA5/FRT/TO-HA-bcTAK1 pcDNA5/FRT/TO-HA-bcIRF7 pcDNA5/FRT/TO-HA-Ub	Immunoblotting, Immunofluorescence Microscopy, Luciferase report assay, Plaque assay and Quantitative Real-time PCR
Luciferase Reporter Assay	
Luci-bcIFNa Luci-DrIFNϕ1 Luci-DrIFNϕ3 Luci-NF-κB pRL-TK	Black Carp IFNa, Zebrafish IFNϕ1/3 and Human NF-κB Promoter Activity Analysis
Knock-Down	
pLKO.1-sh-scramble pLKO.1-sh-bcPRKX-1 pLKO.1-sh-bcPRKX-2 pLKO.1-sh-bcPRKX-3 pLKO.1-sh-bcPRKX-4	Knock-Down bcPRKX in MPK cells

**Table 2:** Antibodies used in this study.

Antibody	Source
Mouse monoclonal antibodies	
anti-Flag anti-HA anti-actin	F2555; Sigma, USA H3663; Sigma, USA A5441; Sigma, USA
Rabbit monoclonal antibodies	
anti-Lamin B1 anti-Histone H3	T40003; Abmart, China T56587; Abmart, China
Rabbit polyclonal antibodies	
anti-HA anti-Myc anti-LAMP1	H6908; Sigma, USA AV38156; Sigma, USA 21997-1-AP; Proteintech, China
Fluorescence secondary antibodies	
anti-mouse IgG H&L (Alexa Fluor 488) anti-rabbit IgG H&L (Alexa Fluor 594)	ab150113; Abcam, UK ab150080; Abcam, UK

**Table 3:** Reagents used in this study.

Reagent	Source
Lipomax Polyethylenimine (PEI) Mouse anti-Flag protein A/G agarose beads Mouse anti-HA protein A/G agarose beads protein A/G agarose beads Chloroquine (CQ) MG-132	32012, SUDGEN, China 24765, polysciences, USA A2220; Sigma, USA A2095; Sigma, USA 36403ES25; Yeasen, China HY-17589A, MCE, USA HY-13259, MCE, USA

### Cloning of PRKX in black carp

To amplify the CDS of *bcPRKX* gene, the primers based on the *bcPRKX* sequence from the transcriptome database were designed, which were listed in **Table 4**. The total RNA of the black carp spleen was isolated by using Trizol (TaKaRa, Japan), and the first-strand cDNA was synthesized by using the PrimscriptTM RT Reagent Kit with gDNA Eraser (TaKaRa, Japan). The CDS of *bcPRKX* gene was cloned into the pMD18-T vector (TaKaRa, Japan) and sequenced by TSINGKE (China).

**Table 4:** Primers used in this study.

Primer	Sequence (5’-3’)	Application
CDS		
bcPRKX-F bcPRKX-R	ATGGCATCAACTAAAGGCAAA TCAGAAGTTTTTGAAAATTTC	Cloning of bcPRKX
Vector Construction		
bcPRKX-F bcPRKX-R	ACTGACGGTACCATGGCATCAACTAAAGGC ACTGACCTCGAGTCAGAAGTTTTTGAAAATTTCCAGA	pcDNA5/FRT/TO-Flag/HA/Myc-bcPRKX construction
qPCR		
bcPRKX-qF bcPRKX-qR bcMXI-qF bcMXI-qR bcViperin-qF bcViperin-qR bcPKR-qF bcPKR-qR bcIκB-b-qF bcIκB-b-qR bcIL-12-qF bcIL-12-qR bcβ-actin-qF bcβ-actin-qR	TAAAGCAGGAGCAACACG TTCACCGCCATTCACATA TGAGCGTAGGCATTAGCAC CCTGGAGCAGCAGATAGCG CCAAAGAGCAGAAAGAGGGACC TCAATAGGCAAGACGAACGAGG GAGCGGACTAAAAGGACAGG AAAATATATGAGACCCAGGG ACCCCTTCCTCAACATAC TACCACAGTCATCCACCA GCAGTTTCTTTCCTCTCTCCT TGTCTCGTTGGTTTCCATTTT TGGGCACCGCTGCTTCCT TGTCCGTCAGGCAGCTCAT	Detecting the corresponding genes expression by qPCR
shRNA		
bcPRKX-shRNA-1-F bcPRKX-shRNA-1-R bcPRKX-shRNA-2-F bcPRKX-shRNA-2-R bcPRKX-shRNA-3-F bcPRKX-shRNA-3-R	CCGGGCAGGAGCAACACGTACATAACTCGAGTTATGTACGTGTTGCTCCTGCTTTTTG AATTCAAAAAGCAGGAGCAACACGTACATAACTCGAGTTATGTACGTGTTGCTCCTGC CCGGGCGGTGAACTCTTCAGCTATTCTCGAGAATAGCTGAAGAGTTCACCGCTTTTTG AATTCAAAAAGCGGTGAACTCTTCAGCTATTCTCGAGAATAGCTGAAGAGTTCACCGC CCGGGCTCATCTTTGAAATGCTAGCCTCGAGGCTAGCATTTCAAAGATGAGCTTTTTG AATTCAAAAAGCTCATCTTTGAAATGCTAGCCTCGAGGCTAGCATTTCAAAGATGAGC	pLKO.1-sh bcPRKX construction

### Phylogenetic analysis of PRKX

Nucleotide sequences of 68 PRKX homologues were retrieved and found on BLAST (GenBank accession numbers are listed in **Supplementary Figure 2**). These species were classified into 5 categories—primates (Primates (12)), rodents (Glires (12)), birds (Aves (12)), reptiles (Reptilia (12)), amphibians (Amphibia (8)) and fish (Teleostei (12)). These nucleotide sequences were input into MEGA 7.0 and multiple sequence alignment was performed by ClustalW. According to the alignment, the phylogenetic tree was constructed with the neighbor-joining method with default parameters.

### Virus production and titration

Grass carp reovirus (GCRV, strain: GCRV873) and spring viremia of carp virus (SVCV, strain: SVCV741) were kept in the lab. GCRV and SVCV were propagated in CIK and EPC independently in the presence of 2% FBS at 26°C. Virus titers were evaluated through the plaque assay in EPC cells as previously described (32). Briefly, the virus was applied to 10-fold serial dilution and the array of the diluted virus was added to EPC cells. After 2 h incubation, the media were changed with fresh DMEM containing 2% FBS and 0.75% methylcellulose (Sigma, USA). And the plaques were measured at day 3 post-infection (32).

### LPS and poly (I:C) treatment

Lipopolysaccharide (LPS) and polyinosinic-polycytidylic acid (Poly (I:C)) were both used for the treatment of MPK cells. LPS was bought from Merck (Germany) and Poly (I:C) was purchased from MedChemExpresss (MCE) (USA). LPS or Poly (I:C) was dissolved in PBS and was prepared as 10 mg/mL. LPS was added directly into the medium with a final concentration of 1, 10 or 50 μg/mL separately; and Poly (I:C) was directly added into the medium as well with a concentration of 5, 25 or 50 μg/mL separately.

### shRNA design

Per pairs of shRNA oligos (listed in **Table 4**) targeting the CDS of *bcPRKX* gene were designed according to *In vivo *Gen siRNA Wizard Software 3.1 (https://www.invivogen.com/sirnawizard/guidelines.php) and were sub-cloned into pLKO.1-TRC as previously (33).

### quantitative real-time PCR

MPK cells were seeded in a 6-well plate (2 × 10^6^ cells/well) at 16 h before treatment. The cells were stimulated with LPS and Poly (I:C) at the indicated concentrations respectively, or infected with GCRV and SVCV at a certain multiplicity of infection (MOI) separately. Then the relative mRNA level of *bcPRKX* gene was examined by quantitative real-time PCR (qPCR) in the Applied Biosystems QuantStudio 5 Real-Time PCR Systems (Thermo Fish, USA) using primers specific to *bcPRKX* gene (**Table 4**). The qPCR program was: denaturation at 95°C/10 min, followed by 40 cycles of 95°C/15 s, 60°C/1 min, followed by dissociation curve analysis (60°C-95°C) to verify the type of product. The relative expression changes of the *bcPRKX* gene among different treatments and different concentrations of stimuli were calculated by the 2^-△△CT^ method (34).

### Subcellular fraction

HEK 293T or EPC cells in a 6-well plate transfected with bcPRKX (3 μg) were harvested at 48 h post-transfection. The harvested cells were incubated in ice-cold Nonidet I P-40 (NP-40) buffer (50 mM Tris-HCl, 150 mM NaCl, 1% NP-40, 5 mM EDTA, 0.1% protease inhibitor cocktail, pH 7.4) for 20 min, then the cells were centrifugated (2700 rpm/4°C) for 5 min. The supernatant media was collected as cytoplasm extract and the sediment was used as nuclei fraction.

### Lysosome isolation

HEK 293T cells in a 6-well plate were transfected with bcTAK1 (1 μg/well) together with bcPRKX (2 μg/well) or empty vector (2 μg/well). The HEK 293T cells were harvested at 48 h post-transfection. The harvested cells were used for lysosome extraction by using the lysosome isolation kit (BB-3603; Bestbio, China). Briefly, the cells were centrifugated at 500 × *g*/4°C for 5 min and resuspended in ice-cold PBS. The cells were incubated with Buffer A for 10 min and then homogenized. The homogenate was centrifugated at 1000 × *g*/4°C for 5 min, 3000 × *g*/4°C for 10 min and 5000 × *g*/4°C for 10 min respectively; and the supernatant was collected for the next step after every centrifugation above. Then the supernatant was centrifugated at 20000 × *g*/4°C for 20 min. The pellet was collected and resuspended in Buffer B, then centrifugated at 20000 × *g*/4°C for another 20 min. The sediment was used as lysosome fraction.

### Immunoblotting (IB)

Total proteins were extracted by lysing EPC or HEK 293T cells in lysis buffer (50 mM Tris-HCl, 150 mM NaCl, 5 mM EDTA, 1% NP-40, pH 7.4). The whole cell lysate was separated by 10% SDS-PAGE and transferred to PVDF membranes (Millipore, USA). Then the membrane was incubated with the primary antibodies after blocking with 5% non-fat milk in TBS (100 mM Tris-HCl, 150 mM NaCl, pH 7.4). After three times of wash with TBST (0.1% Tween-20 in TBS), the membrane was incubated with the corresponding secondary antibodies. Finally, the target proteins on the PVDF membrane were visualized by BCIP/NBT Alkaline Phosphatase Color Development Kit (Sigma, USA).

### Immunofluorescence microscopy

EPC cells in a 24-well plate were transfected with bcTAK1 (200 ng/well) and bcPRKX (300 ng/well). The EPC cells were fixed with 4% (v/v) paraformaldehyde for 10 min and permeabilizated with triton X-100 (0.2% in PBS) for 10 min. Then the cells were incubated with anti-Flag antibody or anti-HA antibody after blocking with 10% bovine serum albumin for 60 min. Subsequently, the cells were incubated with the corresponding secondary antibody including Alexa 594 and Alexa 488; DAPI was used for nucleus staining (34). Finally, the cells were mounted and visualized under a confocal microscope (Olympus).

### Luciferase reporter assay

EPC cells in 24-well plate were co-transfected with bcPRKX (5 ng, 15 ng or 25 ng/well), Luci-bcIFNa (250 ng/well), Luci-DrIFNϕ1/3 (250 ng/well), pRL-TK (25 ng/well), bcTAK1 (25 ng/well) and bcIRF7 (50 ng/well). HEK 293T cells in a 24-well plate were co-transfected with bcPRKX (5 ng, 15 ng or 25 ng/well), Luci-NF-κB (250 ng/well), and pRL-TK (25 ng/well). For each transfection, the total amount of DNA was balanced with the empty vector. The cells were harvested at 24 h post-transfection and lysed for 15 min on ice. Centrifuged supernatant was used to measure the activities of Firefly luciferase and Renilla luciferase according to the manufacturer’s protocol (Promega, USA) (34).

### Co-Immunoprecipitation (co-IP)

HEK 293T cells (in a 10 cm diameter petri dish) were harvested and lysed for co-IP assay at 48 h post-transfection as previously (34). Briefly, the cells were lysed and centrifuged at 12000 rpm for 15 min. Then the centrifuged supernatant was incubated with protein A/G agarose beads at 4°C for 2 h. After the pre-clean, the supernatant was incubated with anti-Flag/HA antibody-conjugated protein A/G agarose beads at 4°C for 4 h. After 3-5 times of washing with 1% NP-40 buffer, the beads were boiled in 5 x sample buffer and used for immunoblotting assay as mentioned above.

### Statistics analysis

qPCR, luciferase reporter assay and virus titration assay were conducted in triplicate. Error bars represent the standard error of the mean value (+SEM) of three independent experiments. The data were analyzed by two-tailed Student’s t-test. Asterisk (*) stands for p<0.05.

## Results

### Cloning and sequencing analysis of bcPRKX

**Figure 1:**
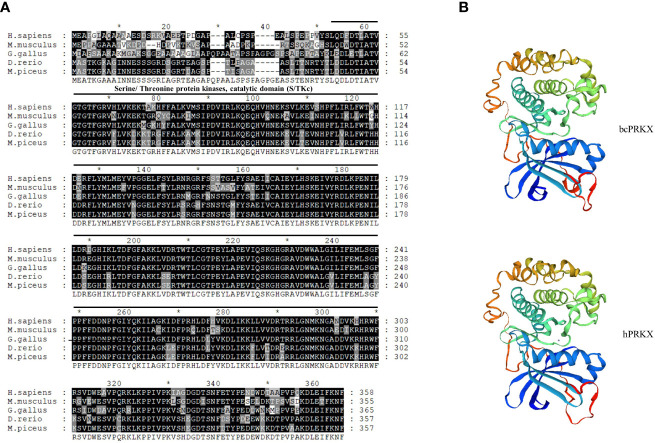
PRKX of black carp (bcPRKX) is conserved among species. **(A)** Amino acid sequences of PRKX from 5 species were aligned by using GeneDoc, including human (*H. sapiens*), mouse (*M. musculus*), chicken (*G. gallus*), zebrafish (*D. rerio*) and black carp (*M. piceus*). **(B)** Comparison between the three-dimension structures of human PRKX and bcPRKX. These models were built by using SWISS-MODEL (SWISS-MODEL (expasy.org)).

**Figure 2:**
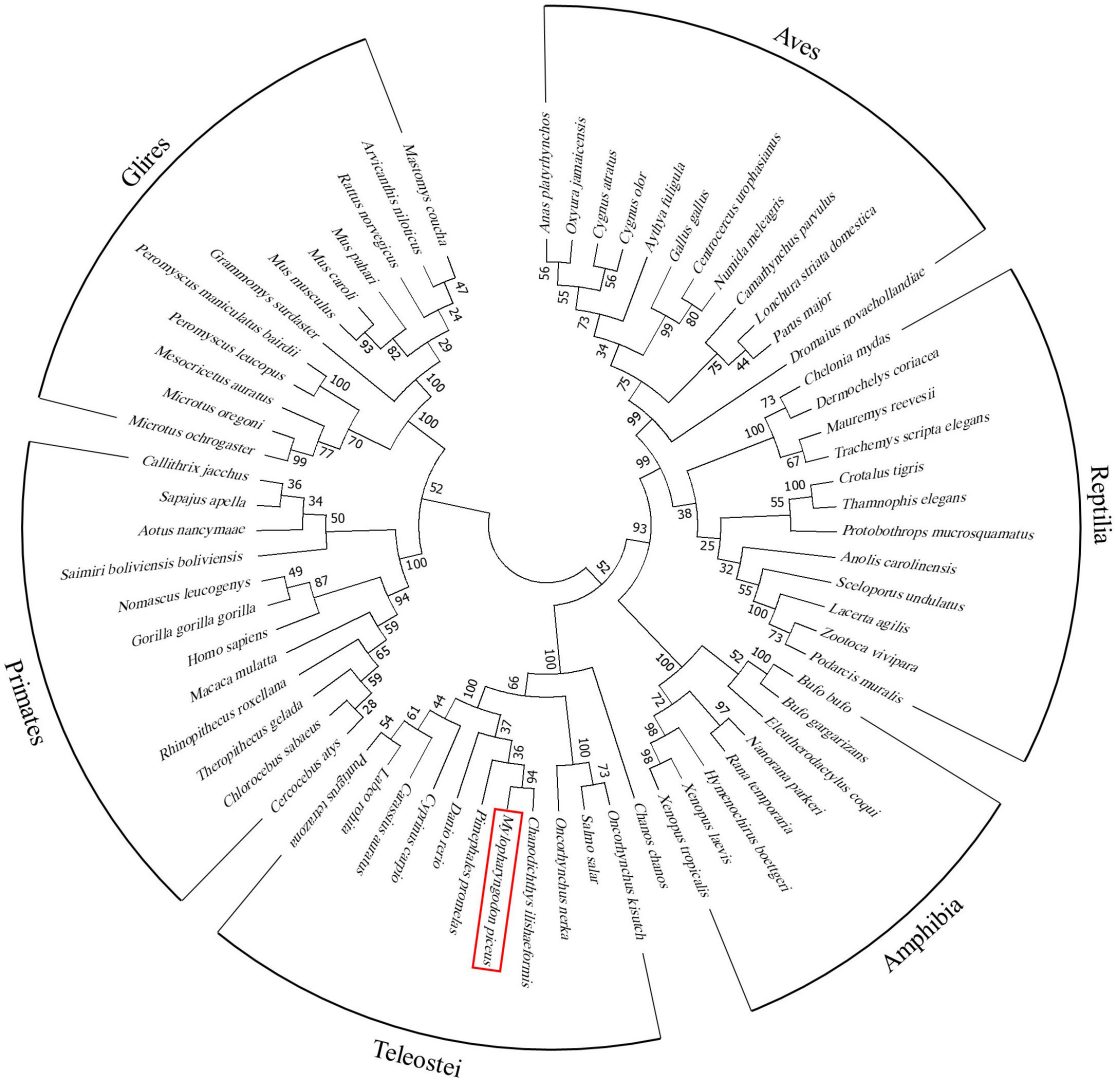
Phylogenetic study of bcPRKX. Nucleotide sequences of 68 PRKX homologues were aligned (GenBank accession numbers are listed in **Supplementary Figure 2**). These species were classified into primates (Primates (12)), rodents (Glires (12)), birds (Aves (12)), reptiles (Reptilia (12)), amphibians (Amphibia (8)) and fish (Teleostei (12)). The result of alignment was built into a phylogenetic tree by using MEGA 7.0.

To investigate the role of PRKX in teleost, bcPRKX was cloned from the black carp spleen. The full-length CDS of *bcPRKX* gene (NCBI accession number: MZ514850) comprises 1074 nucleotides and encodes 357 amino acids (**Supplementary Figure 1**). The predicted theoretical isoelectric points (PI) and molecular mass of bcPRKX are 8.51 and 40.7 kDa respectively (Calculated by EXPASy Compute PI/Mw). To investigate the conservation of bcPRKX, amino acid sequences of PRKX of diverse species have been subjected to multiple alignments, including PRKX of human (*Homo sapiens*), mouse (*Mus musculus*), chicken (*Gallus gallus*) and zebrafish (*Danio rerio*). PRKX is conserved among these above species, especially the Serine/Threonine protein kinases (S/TKc) domain (amino acids 48 to 302) (**Figure 1A**). The protein structure of bcPRKX and human PRKX (hPRKX) were predicted by SWISS-MODEL (https://www.swissmodel.expasy.org/). Consistent with amino acid sequences alignment, the predicted structures of these two proteins were similar (**Figure 1B**). To further elucidate the evolution of PRKX in vertebrates, the phylogenetic tree was constructed based on the PRKX nucleotide sequences from the selected species, in which bcPRKX clustered closely with PRKX of *Chanodichthys ilishaeformis* (**Figure 2**).

### bcPRKX expression *Ex vivo* in response to different immune stimuli

**Figure 3:**
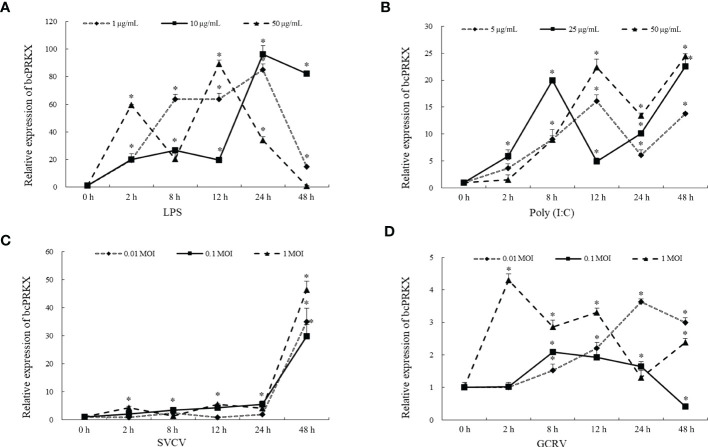
bcPRKX mRNA expression in response to different stimuli. MPK cells were treated with **(A)** LPS (1, 10 and 50 μg/mL), **(B)** Poly (I:C) (5, 25 and 50 μg/mL), **(C)** SVCV (0.01, 0.1 and 1 MOI) or **(D)** GCRV (0.01, 0.1 and 1 MOI) respectively. The cells were harvested at 0, 2, 8, 12, 24 or 48 h post-treatment separately, then subject to RNA isolation and qPCR analysis. Asterisk (*) stands for statistical difference (p<0.05).

To investigate the role of bcPRKX during host innate immune response, MPK cells were subjected to treatment with LPS, Poly (I:C), SVCV or GCRV separately, and the mRNA profile of *bcPRKX* gene within 48 h post-stimulation was examined by qPCR. In the LPS-treated MPK cells, compared to the control group, the transcription of *bcPRKX* was increased right after treatment, then decreased at 8 h (50 μg/mL) or 12 h (10 μg/mL and 1 μg/mL), and increased to the highest level at 12 h (50 μg/mL) or 24 h (10 μg/mL and 1 μg/mL) (**Figure 3A**). In the Poly (I:C)-treated MPK cells, the transcription of *bcPRKX* was increased right after stimulation and varied in a similar way for all three groups (5 μg/mL, 25 μg/mL and 50 μg/mL) However, the variation of group of 25 μg/mL was faster in comparison with the other two (5 μg/mL and 50 μg/mL) (**Figure 3B**). After SVCV infection, the mRNA levels of *bcPRKX* in all three groups (0.01, 0.1 and 1 MOI) increased in a similar way: increased slightly after infection and elevated robustly from 24 h to 48 h (**Figure 3C**). In contrast, the mRNA profiles of *bcPRKX* in MPK cells after GCRV infection were different in three groups (0.01, 0.1 and 1 MOI). The expression of *bcPRKX* gene reached the maximum value at 2 h in 1 MOI group, 0.1 MOI at 8 h and 0.01 MOI at 24 h, which suggested that the higher dosage of GCRV induced the earlier burst of *bcPRKX* gene expression (**Figure 3D**). The data demonstrated that two major RNA viruses threatening the fresh water aquaculture industry in China, GCRV (double-strand RNA virus) and SVCV (single strand RNA virus), initiated host innate immune activation differently. Thus, bcPRKX was involved in host innate immunity triggered by bacteria and viruses but functioned in different mechanisms.

### Protein expression and subcellular distribution of bcPRKX

**Figure 4:**
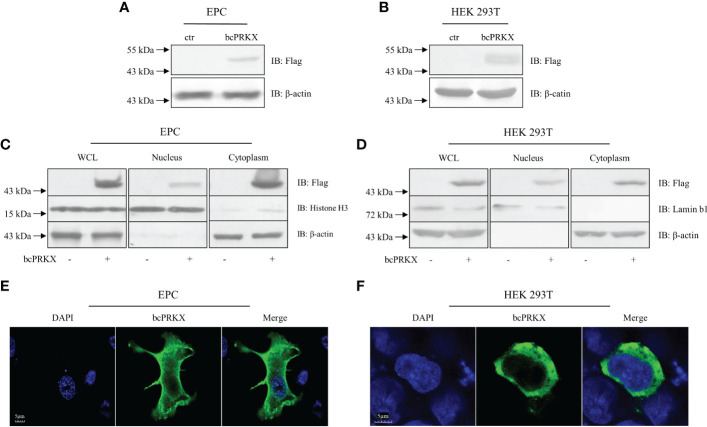
The subcellular distribution of bcPRKX. **(A, B)** EPC or HEK 293T cells in 6-well plates were transfected with bcPRKX. The cells were harvested and lysed for immunoblotting (IB) at 48 h post-transfection. **(C, D)** EPC or HEK 293T cells in 6 cm diameter petri dish were transfected with bcPRKX and subjected to subcellular fraction assay at 48 h post-transfection. The whole cell lysate (WCL), nuclear extract and cytosolic extract were made separately according to the methods. IB for Histone H3 and Lamin b1 were used for nuclear internal reference of EPC and HEK 293T cells respectively; IB for β-actin was used for cytosolic internal reference. **(E, F)** EPC or HEK 293T cells in 24-well plates were transfected with bcPRKX and cells were subjected to immunofluorescence microscopy at 24 h post-transfection. The scale bar represented 5 μm. bcPRKX: pcDNA5/FRT/TO-Flag-bcPRKX.

In order to investigate the mechanism of bcPRKX in the immunity, EPC and HEK 293T cells were transfected with plasmids encoding bcPRKX separately and used for immunoblotting (IB) assay, which demonstrated that bcPRKX expressed well (migrated around 50 kDa and matched the predicted molecule weight) in both fish and mammalian systems (**Figures 4A, B**). To explore the subcellular distribution of bcPRKX, EPC or HEK 293T cells were transfected with plasmids expressing bcPRKX and harvested for the cell fraction analysis, in which bcPRKX was detected in both cytosolic and nuclear extract (**Figures 4C, D**). And the subsequential immunofluorescent staining showed clearly that green fluorescence representing bcPRKX molecules was scattered in the whole cell and mainly in the cytosolic area (**Figures 4E, F**), which matched the data of subcellular fraction. All the above data suggested this black carp PRKX homologue functioned in both cytoplasm and nuclei.

### bcPRKX-regulated IFN and NF-κB signaling

**Figure 5:**
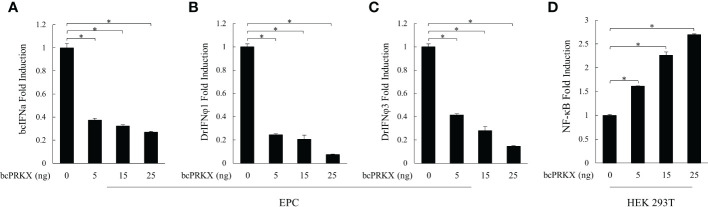
bcPRKX functioned in IFN and NF-κB signaling. **(A-C)** EPC cells were co-transfected with bcPRKX, Luci-bcIFNa or Luci-DrIFNϕ1/3 and pRL-TK separately. **(D)** HEK 293T cells were co-transfected with bcPRKX, Luci-NF-κB and pRL-TK. The cells were harvested for luciferase reporter assay at 24 h post-transfection according to the methods. Asterisk (*) stands for statistical difference (p<0.05).

Mammalian TAK1 is recruited in a number of signaling pathways, such as TGF-β/BMP, Wnt/Fz, JNK, MAPK and NF-κB pathways (15, 16, 35–37). Our previous study has demonstrated that bcTAK1 positively regulates IRF7/IFN signaling (32). The homology among PRKX proteins was very high in the selected species (**Figure 1**), implying the conservation of this molecule in signaling transduction from teleost to human, especially in teleost fishes. To investigate the role of bcPRKX in IFN and NF-κB signaling, EPC or HEK 293T cells were transfected with bcPRKX and used for dual-luciferase report assay separately. The results showed that the transcriptions of the promoters of teleost IFNs, including black carp IFNa (bcIFNa), zebrafish IFNφ1 (DrIFNφ1) and zebrafish IFNφ3 (DrIFNφ3), were all suppressed by bcPRKX in a dose-dependent manner (**Figures 5A–C**). However, the fold induction of NF-κB promoter by bcPRKX was 1.6 (5 ng), 2.3 (15 ng) or 2.7 (25 ng) times to that of the control respectively, which indicated that the transcription of NF-κB promoter was enhanced by bcPRKX in a dose-dependent manner (**Figure 5D**). Thus, the reporter assay data suggested that bcPRKX functioned as a negative regulator in IFN signaling but a positive regulator in NF-κB signaling.

### bcPRKX-regulated antiviral activity

**Figure 6:**
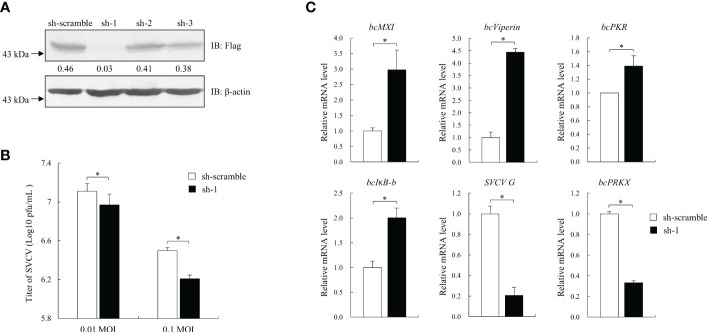
Knock-down of bcPRKX enhanced the cells’ resistance against SVCV. **(A)** HEK 293T cells in a 6-well plate were co-transfected with Flag-bcPRKX and pLKO.1-sh-bcPRKX-1/2/3 (**Table 1**) (or pLKO.1-sh-scramble) respectively. The cells were lysed for immunoblotting at 24 h post-transfection. The numbers represented the densities of bcPRKX bands (normalized to β-actin bands). **(B, C)** MPK cells in a 6-well plate were transfected with pLKO.1-sh-scramble or pLKO.1-sh-bcPRKX-1 and the cells were infected with SVCV at 24 h post-transfection. The media of the cells were harvested for the viral titration at 48 h post-transfection and the cells were used for RNA isolation and qPCR analysis. Asterisk (*) stands for statistical difference (p<0.05).

To further investigate the role of bcPRKX in antiviral signaling, shRNAs targeting CDS of *bcPRKX* gene were designed and the knock-down efficiency of them was analyzed, in which a 94% reduction of bcPRKX protein level was seen when bcPRKX was co-expressed with sh-bcPRKX-1 (**Figure 6A**). MPK cells were transfected with sh-bcPRKX-1 or the control shRNA (sh-scramble) separately and subjected to SVCV infection (0.01 and 0.1 MOI). The plaque assay showed that the virus titers in the media of MPK cells transfected with sh-bcPRKX-1 were lower than that of control cells (**Figure 6B**), which suggested that bcPRKX knock-down led to the improved antiviral activity of host cells. At the same time, the mRNA levels of ISGs in the above MPK cells (0.01 MOI group) were measured and compared by qRT-PCR. The transcription of *bcMX1*, *bcViperin* and *bcPKR* in the bcPRKX knock-down cells was increased 2.9, 4.4 and 1.3 times to those of control cells respectively, and the expression of *bcIκB-b*, the inhibitor of black carp NF-κB, was increased 2 times (**Figure 6C**), which matched the above reporter assay data (**Figure 5**). What is more, the mRNA level of SVCV encoded G protein was much lower in the bcPRKX knock-down cells, which implied that knock-down bcPRKX enhanced host IFN signaling and dampened the replication of SVCV.

### bcPRKX suppressed bcTAK1/bcIRF7 signaling

**Figure 7:**
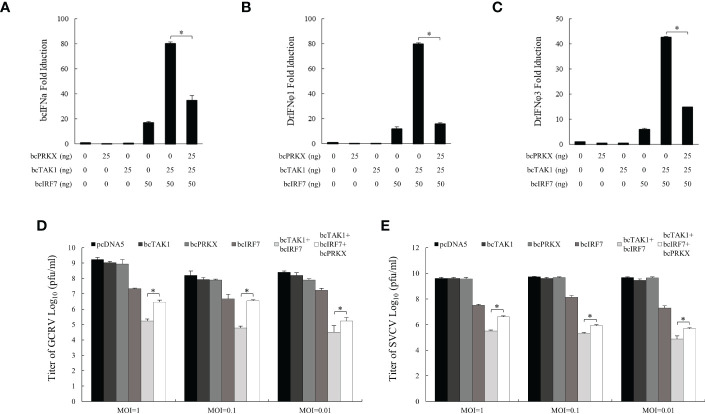
bcPRKX suppressed bcTAK1/bcIRF7/IFN signaling. **(A-C)** EPC cells were co-transfected with Luci-bcIFNa or DrIFNϕ1/3, pRL-TK, bcTAK1, bcIRF7 and/or bcPRKX respectively. At 24 h post-transfection, the cells were subjected to luciferase reporter assay according to the methods. **(D, E)** EPC cells were transfected with bcPRKX, bcTAK1 or bcIRF7 separately, or co-transfected with bcTAK1 and bcIRF7, with or without bcPRKX. At 24 h post-transfection, the cells were infected with SVCV or GCRV separately, and the media were titrated at 24 h post-infection. Asterisk (*) stands for statistical difference (p<0.05).

Studies in mammals and humans have identified PRKX as a significant activator upstream of TAK1 (25). Studies in black carp have revealed that bcTAK1 enhanced bcIRF7-mediated antiviral activity and bcTAB1, a binding protein and activator of bcTAK1, improved bcTAK1/bcIRF7 signaling (38). In order to unveil the function of bcPRKX in bcTAK1/bcIRF7 signaling cascade, EPC cells were co-transfected with bcPRKX, bcTAK1 and bcIRF7, and used for luciferase reporter assay, in which the activities of bcIFNa, DrIFNφ1 and DrIFNφ3 promoters were examined respectively. Overexpression of bcTAK1 alone cannot activate the IFN promoters. When bcTAK1 and bcIRF7 were co-expressed, bcTAK1 could greatly increase the activation levels of the IFN promoters mediated by bcIRF7. However, bcPRKX dramatically reduced the activities of the IFN promoters that were caused by the bcTAK1/bcIRF7 axis when it was co-expressed with bcTAK1 and bcIRF7. The activation level of bcIFNa, DrIFNφ1 and DrIFNφ3 decreased by ~56%, ~80% and ~65%, respectively (**Figures 7A-C**). To further explore the role of bcPRKX in bcTAK1/bcIRF7-mediated antiviral activity, EPC cells were co-transfected with bcPRKX, bcTAK1 and bcIRF7, and subjected to GCRV or SVCV infection. The plaque assay data showed that the viral titers of EPC cells co-expressing bcTAK1 and bcIRF7 were obviously lower than those of EPC cells expressing bcIRF7 alone, although EPC cells expressing bcTAK1 alone owned similar viral titers to those of control cells. However, the virus titers, of both GCRV and SVCV, in the cells co-expressing bcPRKX, bcTAK1 and bcIRF7 were obviously higher than those in the cells co-expressing bcTAK1 and bcIRF7, which suggested that the enhanced antiviral activity of bcIRF7 by bcTAK1 was suppressed by bcPRKX (**Figures 7D, E**).

### bcPRKX mediated degradation of bcTAK1

**Figure 8:**
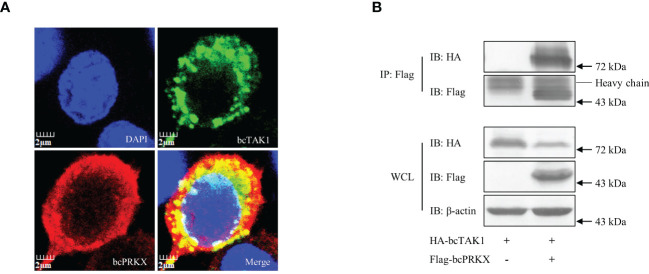
The interaction between bcPRKX and bcTAK1. **(A)** EPC cells were co-transfected with Flag-bcPRKX and HA-bcTAK1 and the cells were fixed for immunofluorescence microscopy at 24 h post-transfection according to the methods. The scale bar represented 2 μm. **(B)** HEK 293T cells in a 10 cm diameter petri dish were co-transfected with bcPRKX and/or bcTAK1 separately. The cells were harvested for immunoprecipitation (IP) assay and subsequential immunoblot (IB) assay at 48 h post-transfection according to the methods.

To explore the mechanism behind the negative regulation of bcTAK1/bcIRF7 signaling by bcPRKX, the interaction between bcPRKX and bcTAK1 was studied through immunofluorescence (IF) microscopy and co-immunoprecipitation (co-IP) analysis. In the IF data, the bcPRKX-expressing region (red color) overlapped with the bcTAK1-expressing region (green color), which implied a similar subcellular distribution of these two molecules (**Figure 8A**). In the co-IP data, bcTAK1 was precipitated by anti-Flag antibody conjugated protein A/G agarose beads incubated with the protein extract from the cells co-expressing HA-bcTAK1 and Flag-bcPRKX, but not from the cells expressing HA-bcTAK1 alone (**Figure 8B**), suggesting the association between these two molecules.

**Figure 9:**
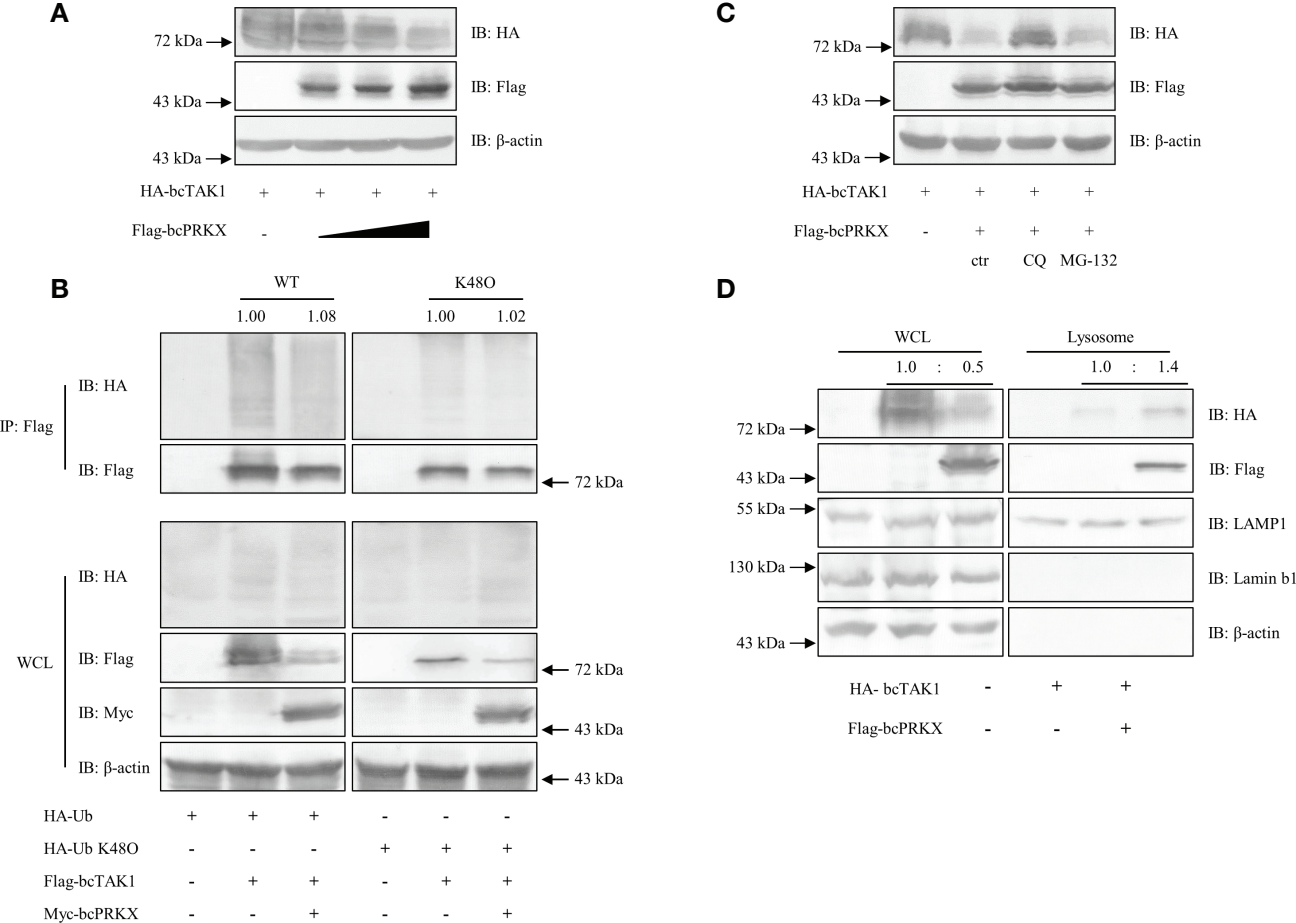
bcPRKX-mediated degradation of bcTAK1. **(A)** HEK 293T cells in a 6-well plate were co-transfected with bcTAK1 and bcPRKX (with the increasing dosage). The transfected cells were harvested for immunoblotting at 48 h post-transfection. **(B)** HEK 293T cells in a 10 cm diameter petri dish were co-transfected with HA-Ub, Flag-bcTAK1 and/or Myc-bcPRKX separately. The transfected cells were harvested for immunoprecipitation and immunoblotting at 48 h post-transfection. **(C)** HEK 293T cells in a 6-well plate were co-transfected with bcTAK1 and bcPRKX and the transfected cells were subjected to chloroquine (CQ) or MG-132 treatment at 8 h before being harvested for immunoblot. **(D)** HEK 293T cells in a 6-well plate were transfected with bcTAK1 alone or co-transfected with bcTAK1 and bcPRKX. The cells were harvested at 48 h post-transfection for lysosome isolation and immunoblotting. IB for LAMP1 was used for lysosomal internal reference.

To further explore the relationship between bcTAK1 and bcPRKX, HEK 293T cells and EPC cells were co-transfected with bcTAK1 and bcPRKX separately, and the impact on the protein level of bcTAK1 by bcPRKX was examined by immunoblot assay. The data in both HEK 293T cells (**Figure 9A**) and EPC cells (**Supplementary Figure 3**) showed with the addition of bcPRKX, the protein level of bcTAK1 decreased in a dose-dependent manner, suggesting that bcPRKX could trigger the degradation of bcTAK1. Next, to examine whether the ubiquitination level of bcTAK1 was affected by bcPRKX, HEK 293T cells were co-transfected with HA-Ub or HA-Ub-K48O, bcTAK1 and/or bcPRKX, then used for immunoprecipitation and immunoblot assay. The bcTAK1 ubiquitination level in the cells expressing bcTAK1 alone was similar to that in the cells co-expressing bcTAK1 and bcPRKX. Besides, the K48-linked ubiquitination level of bcTAK1 in the cells expressing bcTAK1 alone showed no difference from that in the cells co-expressing bcTAK1 and bcPRKX. (**Figure 9B**). These results suggested that bcPRKX-mediated bcTAK1 degradation was independent of the ubiquitin/proteasome pathway. Then, HEK 293T cells were co-transfected with bcTAK1 and bcPRKX, and proteasome inhibitor MG-132 or lysosome inhibitor chloroquine (CQ) was added separately before cell harvest. As shown in the immunoblot data, MG-132 did not impact the induced bcTAK1 degradation by bcPRKX; however, the addition of CQ rescued bcPRKX-mediated bcTAK1 degradation (**Figure 9C**), which implied that bcPRKX triggered the degradation of bcTAK1 through lysosome. To further explore this, HEK 293T cells expressing bcTAK1 alone or co-expressing bcTAK1 and bcPRKX were harvested and used for lysosome extraction separately. The subsequential immunoblot assay showed that bcTAK1 protein level in the whole cell lysate in the bcTAK1 transfection group was obviously higher than that in the bcTAK1/bcPRKX co-expression group. In contrast, in the lysosome, the protein level of bcTAK1 in the bcTAK1 transfection group was considerably lower than that in the bcTAK1/bcPRKX co-expression group, suggesting that bcPRKX was responsible for recruiting bcTAK1 to the lysosome and causing the degradation of bcTAK1 (**Figure 9D**).

## Discussion

As a component of the NF-κB signaling pathway, TAK1 plays an important role in the pro-inflammation response mediated by TNF receptor, IL-1R and TLR. Besides, the positive role of mammalian TAK1 in the IRF3/IFN signaling pathway has been reported (17). Similarly, in our previous study, bcTAK1 has been characterized as an important component in black carp IRF7/IFN signaling cascade, in which bcTAK1 was able to enhance bcIRF7-mediated IFN production, thereby enhancing the resistance of the cells against SVCV and GCRV (32). However, unlike human TAK1, bcTAK1 presented little impact on NF-κB signaling pathway, although amino acid sequence and function domains were conserved between bcTAK1 and human TAK1 (32).

There are some similarities and differences in the regulation of TAK1 between mammals and teleost fish as well. TAK1-binding protein 1 (TAB1) of black carp (bcTAB1), as a chaperone of bcTAK1, unlike its mammalian counterpart, was capable to magnify TAK1/IRF7/IFN signaling but not TAK1/NF-κB signaling during the antiviral innate immune response (38). In mammals, PRKX and PKACα, two upstream kinases of TAK1, associate with and trigger the phosphorylation and activation of TAK1, leading to the activation of MAPK and NF-κB. However, in our previous study, black carp PKACα (bcPKACα) restrained TAK1/IRF7/IFN signaling cascade but played a positive role in the NF-κB pathway (33). In the present study, similar to bcPKACα, bcPRKX functioned as a negative regulator in TAK1/IRF7/IFN axis but a positive regulator in NF-κB signaling (**Figures 5**-**7**). Overexpression of bcPRKX enhanced the transcription of NF-κB promoter (**Figure 5D**) in the luciferase reporter assay, and bcPRKX knockdown led to the enhanced transcription of *bcI-κBb* in host cells (**Figure 6C**), demonstrating the positive role of this molecule in the NF-κB signaling. Since bcTAK1 showed little impact on the NF-κB pathway, it is speculated that bcPRKX associates with other molecules, but not bcTAK1, to take part in the NF-κB signaling, which implied the difference of the regulation in pro-inflammation response between teleost and mammals. Overexpressed bcPRKX was detected in both the nucleus and cytoplasm, which suggested that bcPRKX functioned in both areas (**Figure 4**). However, as a protein with multiple functions, nuclear bcPRKX might play a role in other bioprocesses instead of innate immunity. Some studies have shown that PRKX could translocate into the nucleus and bind to CRE (cAMP-responsive element) promoter, facilitating epithelial cell migration, endothelial cell proliferation and migration in blood vessels (28, 29).

The principal mechanisms for protein degradation include the ubiquitin-proteasome, lysosome, and autophagosome pathways (39). Our data demonstrated that black carp PRKX triggered the lysosome-dependent degradation of TAK1 Interestingly, we found that compared to the cells expressing bcTAK1 alone, a higher protein level of bcTAK1 was detected in the lysosome fraction when co-expressing bcTAK1 and bcPRKX, although the total protein level of bcTAK1 in the whole cell lysate was much less. (**Figure 9D**). Besides, in the lysosome fraction, the protein level of bcPRKX was notably high in comparison to any other proteins. In addition, cytoplastic bcPRKX was distributed relatively evenly (**Figures 4E, F**), which was compatible with the sub-cellular distribution and motility of lysosome (40, 41). Thus, our data implied that bcPRKX might be a lysosomal protein. It is speculated that bcTAK1 was transferred to the lysosome through a certain mechanism and bound to bcPRKX for degradation, which needed to be further explored. Moreover, it has been reported that members of c-AMP-dependent serine/threonine protein kinases family have the potential to incur degradation of certain proteins. For example, a mutant of PKACα, a member of this family, has been shown to trigger the degradation of RIIβ by a caspase-dependent pathway (42). Furthermore, evidence has been shown that the regulation of protein level was an important mechanism in TAK1 regulation. Recently, it was reported that TRIM16 was able to mediate the degradation of phospho-TAK1, therefore ameliorating nonalcoholic steatohepatitis (43). In addition, lysosome-mediated protein degradation is a prevalent manner in regulating the functions of certain proteins in various signaling pathways of teleost fish. It has been reported that zebrafish RNA-binding motif protein 47 (RBM47) promotes MAVS degradation in a lysosome-dependent manner to inhibit IRF3/7 activation and to suppress IFN production, thereby impairing the host antiviral response (44). All these features coincide with the manner how bcPRKX regulated bcTAK1 in innate immune signaling pathway.

In summary, our study has identified the role of bcPRKX in negatively regulating the IFN production. bcPRKX functions as a negative regulator in the bcTAK1/bcIRF7/IFN signaling pathway by inducing the lysosome-dependent degradation of bcTAK1. These results reveal a previously unreported role for PRKX in both teleost fish and mammals and provide new insights for better understanding the regulatory mechanism of innate immunity.

## Data availability statement

The original contributions presented in the study are included in the article/**Supplementary Material**, further inquiries can be directed to the corresponding author/s.

## Author contributions

HF conceived and designed the experiments. YA, XY, LC, CW and JiL performed the experiments. CW, JZ, JuL, HW and JX analyzed the data. XY and HF wrote and reviewed the manuscript. HF and MC supervised the research. All authors contributed to the article and approved the submitted version.

## Supplementary material

Supplementary Figure 1 Nucleotide and amino acid sequences of bcPRKX.

Supplementary Figure 2 Species and accession numbers of PRKX homologues in .

Supplementary Figure 3 EPC cells in a 6-well plate were co-transfected with bcTAK1 and bcPRKX (with the increasing dosage). The transfected cells were harvested for immunoblotting at 48 h post-transfection.
