# Supplementary figures and images for: PRKX down-regulates TAK1/IRF7 signaling in the antiviral innate immunity of black carp Mylopharyngodon piceus
Source: Front Immunol. 2023 Jan 11;13:999219. doi: 10.3389/fimmu.2022.999219 (PMC9875139; doi:10.3389/fimmu.2022.999219)

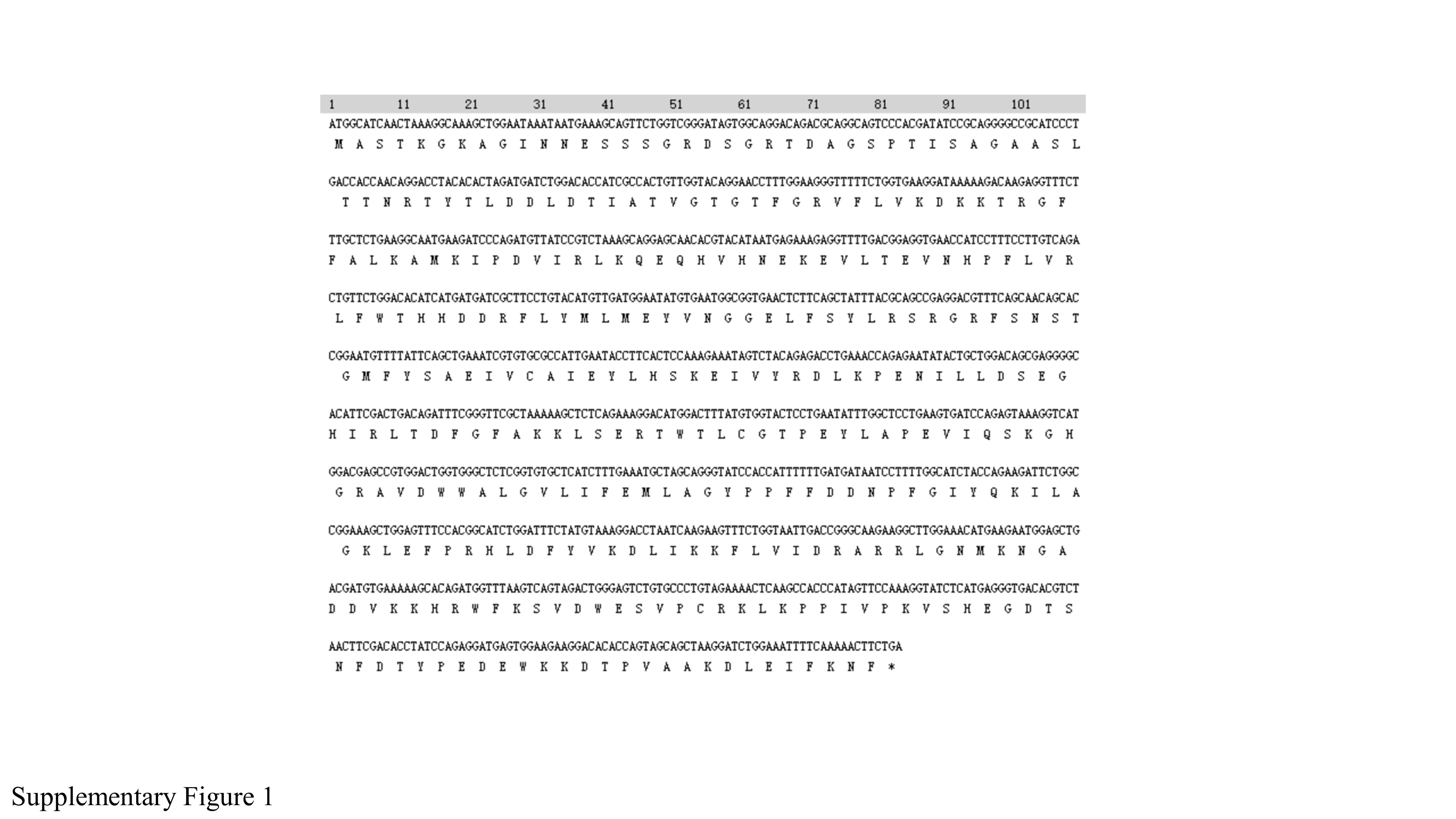

Supplement: Supplementary Figure 1 — Nucleotide and amino acid sequences of bcPRKX. [file Image_1.jpeg]

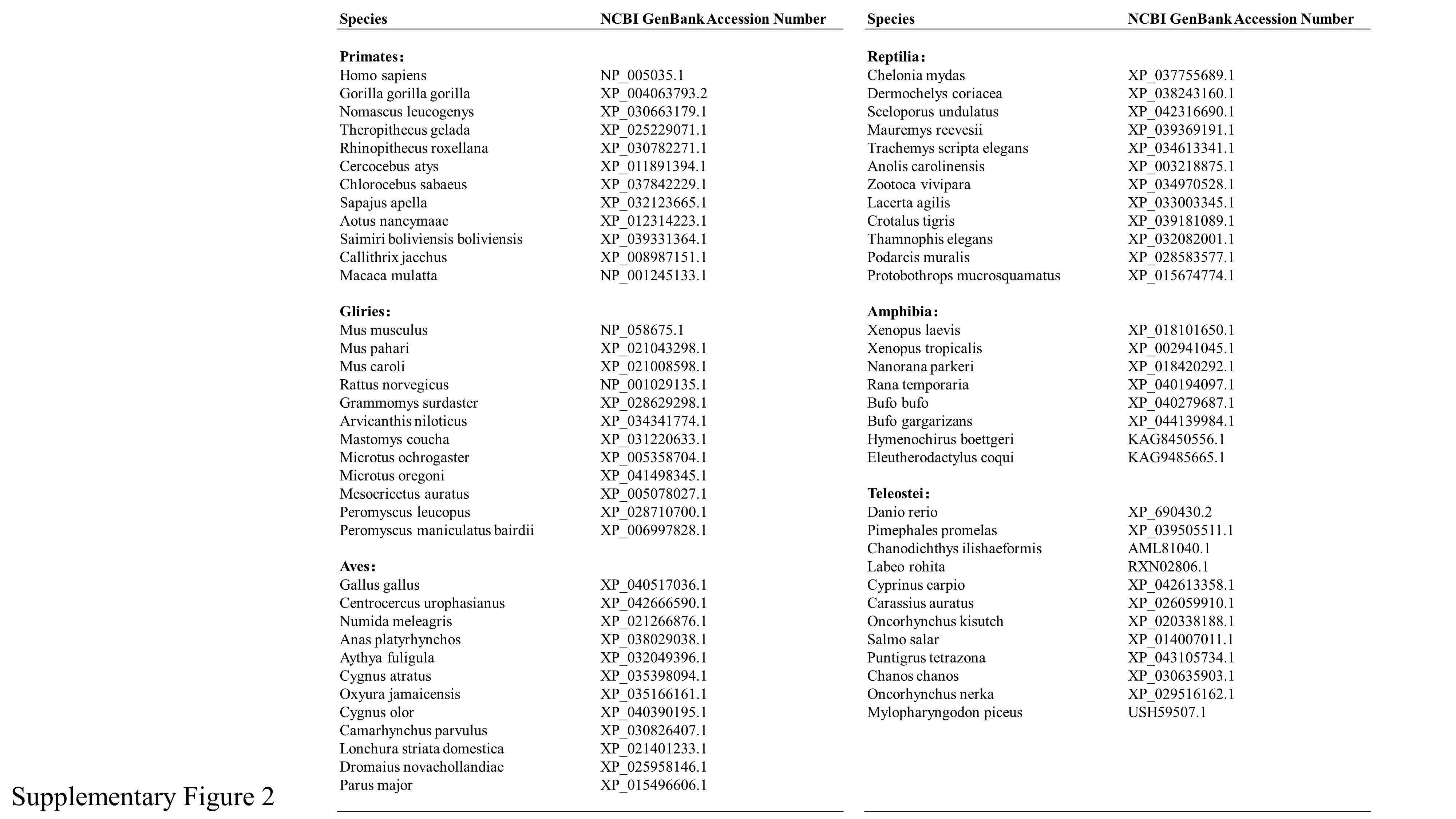

Supplement: Supplementary Figure 2 — Species and accession numbers of PRKX homologues in . [file Image_2.jpg]

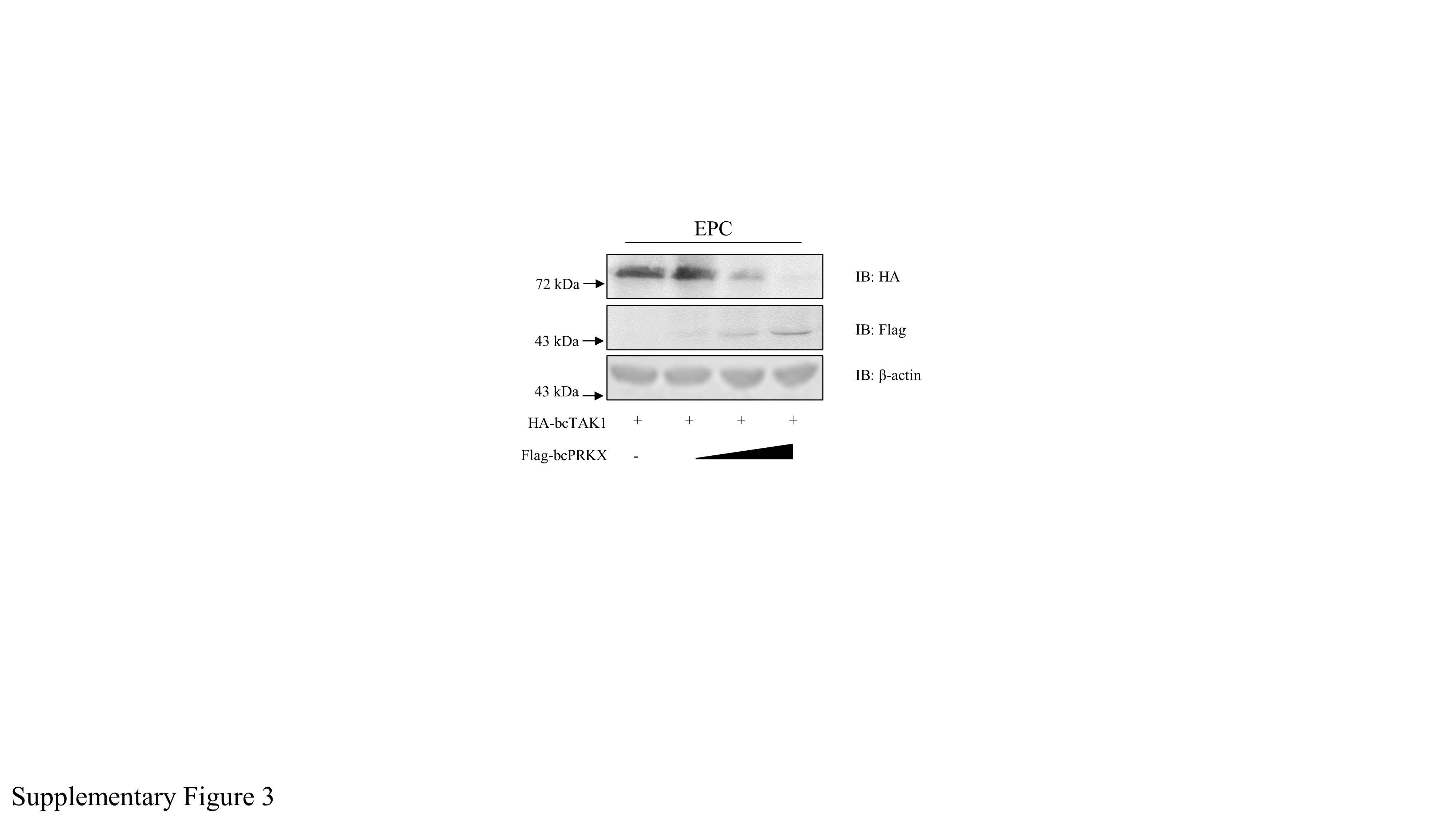

Supplement: Supplementary Figure 3 — EPC cells in a 6-well plate were co-transfected with bcTAK1 and bcPRKX (with the increasing dosage). The transfected cells were harvested for immunoblotting at 48 h post-transfection. [file Image_3.jpeg]
